# A genomic survey of proteases in Aspergilli

**DOI:** 10.1186/1471-2164-15-523

**Published:** 2014-06-25

**Authors:** Sebnem Ozturkoglu Budak, Miaomiao Zhou, Carlo Brouwer, Ad Wiebenga, Isabelle Benoit, Marcos Di Falco, Adrian Tsang, Ronald P de Vries

**Affiliations:** CBS-KNAW Fungal Biodiversity Center, Uppsalalaan 8, Utrecht, 3584 CT The Netherlands; Faculty of Agriculture, Department of Dairy Technology, University of Ankara, Ankara, Turkey; Fungal Molecular Physiology, Utrecht University, Utrecht, The Netherlands; Centre for Structural and Functional Genomics, Concordia University, 7141 Sherbrooke Street West, Montreal, QC H4B 1R6 Canada

## Abstract

**Background:**

Proteases can hydrolyze peptides in aqueous environments. This property has made proteases the most important industrial enzymes by taking up about 60% of the total enzyme market. Microorganisms are the main sources for industrial protease production due to their high yield and a wide range of biochemical properties. Several Aspergilli have the ability to produce a variety of proteases, but no comprehensive comparative study has been carried out on protease productivity in this genus so far.

**Results:**

We have performed a combined analysis of comparative genomics, proteomics and enzymology tests on seven *Aspergillus* species grown on wheat bran and sugar beet pulp. Putative proteases were identified by homology search and Pfam domains. These genes were then clusters based on orthology and extracellular proteases were identified by protein subcellular localization prediction. Proteomics was used to identify the secreted enzymes in the cultures, while protease essays with and without inhibitors were performed to determine the overall protease activity per protease class. All this data was then integrated to compare the protease productivities in Aspergilli.

**Conclusions:**

Genomes of *Aspergillus* species contain a similar proportion of protease encoding genes. According to comparative genomics, proteomics and enzymatic experiments serine proteases make up the largest group in the protease spectrum across the species. In general wheat bran gives higher induction of proteases than sugar beet pulp. Interesting differences of protease activity, extracellular enzyme spectrum composition, protein occurrence and abundance were identified for species. By combining *in silico* and wet-lab experiments, we present the intriguing variety of protease productivity in Aspergilli.

**Electronic supplementary material:**

The online version of this article (doi:10.1186/1471-2164-15-523) contains supplementary material, which is available to authorized users.

## Background

Proteases form a complex family of enzymes that possess different catalytic mechanisms with various active sites and divergent substrate specificities [1, 2]. Proteases hydrolyze peptides in aqueous environments [3, 4] and for years this ability has been utilized in industrial processes like food processing, waste treatment, textiles/detergent applications, and photography/chemical processing [5–9]. Proteases can be classified into four major groups: aspartic, cysteine, metallo and serine proteases [2]. Protease inhibitors for each of these classes have been described [10]. These inhibitors regulate the activity of proteases by binding to the enzyme and eliminating unwanted proteolysis [11, 12]. In recent years, proteases and protease inhibitors have gained additional interests in many health related areas as e.g. pathogenic agents by allergy, asthma and obese related illness [13]. Proteases have been recognized as the most important industrial enzymes accounting for about 60% of the total enzyme market [14].

Proteases can be obtained from animal, plant and microbial sources [7]. However, microorganisms are the most important sources for industrial applications [3, 4] due to their high yield and productivity and a wide range of biochemical and catalytic properties [4]. The genus *Aspergillus* represents a diverse group of filamentous ascomycetous fungi [15], including human, animal and plant pathogens, but also species with a major role in industrial biotechnology [16]. Several *Aspergillus* species have the ability to produce a variety of proteases [17–22].

In this study we have performed a genome survey of several Aspergilli based on the protein sequences of verified proteases and Pfam domains. Curated putative proteases were fed to a combination of protein subcellular localization (SCL) predictors to identify the potentially secreted proteins. The results of this *in silico* comparative secretomics were then tested by enzyme activity assays and proteomic experiments on samples from cultures grown on wheat bran and sugar beet pulp. Protease inhibitors were used to determine the contribution of the various protease classes to the total protease activity. Finally, by combining comparative genomes, proteomics and enzymology tests, we demonstrate the intriguing variety of protease productivity in the Aspergilli.

## Results

### Genome mining and extracellular protein clustering

The genomes of seven *Aspergillus* species, *Aspergillus niger* ATCC 1015 [23], *Aspergillus nidulans* FGSC A4 [24], *Aspergillus oryzae* RIB40 [25], *Aspergillus flavus* NRLL 3357 [26], *Aspergillus terreus* NIH 2624, *Neosartorya fischeri* CBS 544.65 [27] and *Aspergillus fumigatus* AF293 [27] (Table 1, data retrieved from AspGD [28]), were included in the genomic comparison of protease-encoding genes. On the basis of putative protease clusters (588 proteins, 478 clusters) already existing in AspGD, additional putative proteases were found by homology. Gene models were manually corrected by multiple sequence alignments. A thorough Pfam domain detection was carried out on the *Aspergillus* genomes. Proteins containing no known protease-related Pfam domain(s) were removed when no additional literature support could be found. At the end, 1558 extra putative proteases were added to the original set of AspGD protein clusters by Jaccard [29] and OrthoMCL [30] (in total 2146 proteins, 478 clusters) (Additional file 1). While investigating the gene presence/absence patterns, genome scale ortholog clusters were utilized to identify species-specific genes. 236 out of the 478 clusters appeared to be ubiquitous, by containing at least 1 protein from each species. 56 clusters contained only a single member with no homologs in other species, and were therefore considered “orphan genes” [31, 32]. The other clusters cover the species partially (Additional file 2).

**Table 1:** **Summary of putative proteases in Aspergilli**

Species	Genome reference	Total genes	Putative proteases	Serine proteases	Aspartic proteases	Metallo proteases	Amino proteases	Missellaneous proteases
*A. fumigatus* **Af293**	[27]	9781	301 (45)	75 (16)	17 (9)	48 (8)	28 (5)	133 (7)
*A. flavus NRLL* **3357**	[26]	12604	336 (63)	88 (23)	21 (15)	61 (9)	24 (4)	142 (12)
*A. oryzae* **RIB40**	[25]	12030	336 (57)	85 (21)	21 (14)	66 (11)	25 (5)	139 (6)
*A. terreus* **NIH 2624**	Unpublished	10406	306 (44)	73 (16)	18 (9)	58 (6)	26 (4)	131 (9)
*N. fischeri* **CBS 544.65**	[27]	10406	307 (45)	76 (15)	15 (11)	50 (7)	26 (4)	140 (8)
*A. nidulans* **FGSC A4**	[24]	10680	302 (40)	72 (13)	16 (8)	50 (6)	24 (5)	140 (8)
*A. niger* **ATCC 1015**	[23]	11162	314 (53)	84 (22)	19 (15)	57 (7)	25 (3)	129 (6)

The numbers of extracellular proteins are provided in brackets following each category.

Six different protein SCL predictors were applied to all 2146 putative proteases. By using majority vote 335 proteins were considered extracellular, among which 277 were in the original AspGD protease clusters (Additional file 3).

Further classification of proteases was determined by combined manual literature search and Pfam annotations. At the end, most putative proteases were classified into four major groups, namely amino, aspartic, metallo and serine, while the remaining genes formed the miscellaneous group (Additional file 1).

### Effect of wheat bran and sugar beet pulp on extracellular protease induction in *Aspergilli*

**Figure 1:**
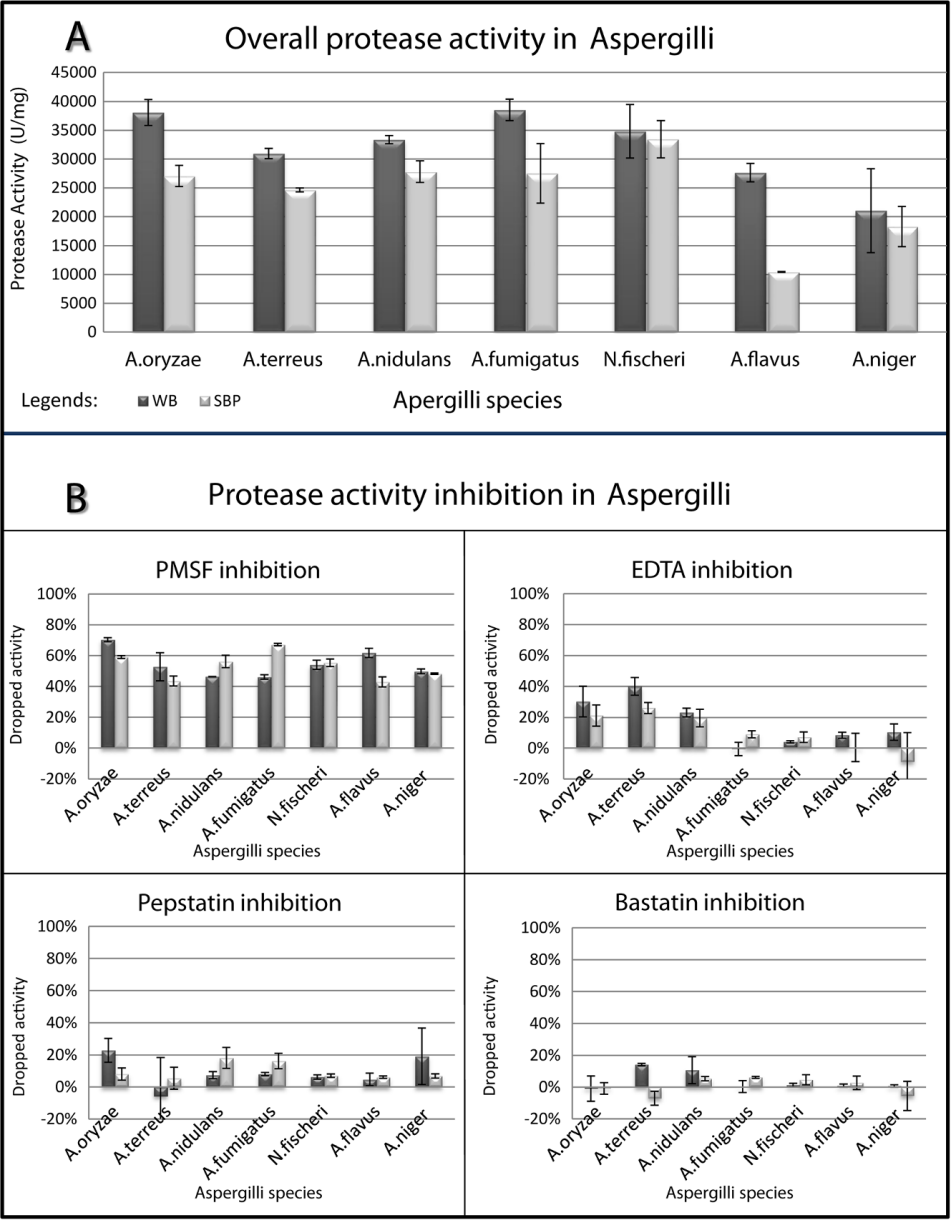
**Protease activity with and without inhibitor in Aspergilli using WB and SBP as carbon sources. A**: Overall protease activity in Aspergilli growing on WB and SBP. Seven *Aspergilllus* species were grown on WB or SBP on 30°C and sampled at 72 h, protease activities were measured for each sample in 2 biological replications with technical triplicates. **B**: Protease activity inhibition in Aspergilli. With the same settings described for Figure 1A, the protease activity was measured after adding corresponding inhibitors. The ratio of dropped activity was calculated by PercentageDroppedActivity = [1- (activity after adding inhibitor/original activity without inhibitor)]%. This dropped activity indirectly represents the proportion of corresponding protease activity in the supernatants, higher this number, bigger proportion of such type of protease takes the overall activity. Legends: WB and SBP: protease activity in wheat bran and sugar beet pulp, respectively.

Two cultivation media, minimal medium with 1% wheat bran (WB) and minimal medium with 1% sugar beet pulp (SBP), were used to induce extracellular protease production in Aspergilli, resulting in an interesting variability of protease activity (Figure 1A). Among the tested species, *N. fischeri* produced the highest protease activity on SBP, *A.fumigatus* produced the highest activity on WB whereas *A. flavus* had the most moderate activities in both substrates. In all cases WB induced more protease activity than SBP. This was particularly true for *A. flavus* and *A. fumigatus*, where the extracellular protease activities on WB were around twice as high as those on SBP. In contrast, for *N. fisheri* only a small difference (<10%) was detected.

### Extracellular proteases in 7 Aspergilli confirmed by proteomics

In order to confirm protease production variability by *Aspergillus* species on different carbon sources, we performed proteomics experiments. In total, 133 putative proteases were identified (at least 2 unique peptides found per protein, Additional file 4). The identified proteases were then mapped to the extended protease clusters created by comparative genomics, resulting in the presence of 45 orthologous groups (OG) in the cultures (Table 2).

**Table 2:** **Putative proteases identified by proteomics in 7 Aspergilli on wheat bran and sugar beet pulp**

Ortholog groups	*A. nidulans*	*A. niger*	*A. oryzae*	*A. terreus*	*A. favus*	*N. fischeri*	*A. fumigatus*	Protease group	Inh.	Ref.
OG-1	AN2572^WS^	185799	-	ATEG_09875^WS^	AFL2G_05512^WS^	NFIA_084570^WS^	Afu2g09030^WS^	Amino	B	[33]
OG-2	AN3918 ^W^	214665^W^	AO090012000110	ATEG_09990	AFL2G_03045	NFIA_032410	Afu2g00220^W^	Amino	B	[34, 35]
OG-3	AN6438^WS^		AO090023000602^W^	ATEG_05269^W^	AFL2G_04433^WS^	NFIA_106690	Afu4g09320^S^	Amino	B	[36]
OG-4	AN8445^WS^	-	AO090003000354^WS^	ATEG_09137^WS^	AFL2G_02631^WS^	NFIA_001250^WS^	Afu3g00650^WS^	Amino	B	[36]
OG-5A	AN1638		AO090023000645	*ATEG_05313* ^W^	AFL2G_04474	NFIA_107140	Afu4g09030	Amino	B	[37]
OG-5B	AN4282	130008	AO090102000639	*ATEG_07980* ^W^	AFL2G_10011	NFIA_037760	Afu5g04330	Amino	B	[36, 38]
OG-6	-	*206384* ^W^	AO090009000228	ATEG_03227	AFL2G_10434	NFIA_049620	Afu6g03260	Aspartic	P	[17, 39]
OG-7A	-	-	-	-	*AFL2G_11784* ^W^	-	-	Aspartic	P	[40]
OG-7B	AN8102	189966	AO090010000644	-	*AFL2G_04852* ^W^	NFIA_002160	Afu3g01220	Aspartic	P	[17, 40]
OG-8	AN4422	213261^WS^	AO090023000872	ATEG_05510^WS^	AFL2G_04683	NFIA_109180	Afu4g07040	Aspartic	P	[34]
OG-9	AN2903^W^	37080	AO090003000693^WS^	ATEG_04676^W^	AFL2G_02316^WS^	NFIA_065900	Afu3g11400^WS^	Aspartic	P	[41]
OG-10	-	40218	-	ATEG_04955^WS^	-	NFIA_065900		Aspartic	P	[17, 40]
OG-11A	-	191956^WS^	AO090009000148	ATEG_05628	AFL2G_10504^W^	NFIA_051920	Afu6g05350	Aspartic	P	[39]
OG-11B	AN6487	53364^WS^	AO090701000002	ATEG_05628	AFL2G_05659	NFIA_051920	Afu6g05350	Aspartic	P	[39]
OG-12	-	211797^WS^	-	-	-	NFIA_100060	-	Aspartic	P	[40]
OG-13	AN6888	201655^WS^	AO090120000474	ATEG_06182	AFL2G_08462	NFIA_073740^WS^	Afu5g13300^WS^	Aspartic	P	[42]
OG-14	AN6796	210782	*AO090012000695* ^W^	ATEG_09753	AFL2G_03570	NFIA_040680	Afu5g01430	Cysteine	E	
OG-15	AN7962^WS^	-	AO090001000135	ATEG_04941^WS^	AFL2G_07373	NFIA_102630^WS^	Afu4g13750^WS^	Metallo	E^d^	[43, 44]
OG-16	-	46803	AO090011000036^WS^	ATEG_07544^WS^	AFL2G_04842^WS^	NFIA_099860^S^	Afu8g07080^WS^	Metallo	E^d^	[45]
OG-17	-	-	AO090010000493^WS^	-	AFL2G_11655^W^	-	-	Metallo	E^d^	[46]
OG-18	-	-	AO090011000052^WS^	-	AFL2G_04856^W^	-	-	Metallo	E^d^	[46]
OG-19	AN10540	-	*AO090023000980* ^WS^	ATEG_06810	-	NFIA_110010	Afu4g06140	Metallo	OG-19	
OG-20	-	209872	AO090005000457	*ATEG_06341* ^W^	AFL2G_00447	NFIA_027170	Afu7g05930	Metallo	OG-20	
OG-21	-	52703^WS^	-	-	AFL2G_03937^WS^	NFIA_033550^WS^	Afu2g01250^WS^	Serine	OG-21	[39, 47]
OG-22A	-	55493^WS^	AO090026000083	-	AFL2G_07153	-	-	Serine	P^m^	[39]
OG-22B	-	55133^WS^	-	-	-	-	-	Serine	P^m^	[39]
OG-23	AN6273	52126	AO090026000357^WS^	ATEG_01242	AFL2G_06902^WS^	NFIA_087760	Afu2g12630	Serine	P^m^	[48]
OG-24	-	46979^WS^	AO090020000351	ATEG_07509	AFL2G_10957	NFIA_047470	Afu6g00310	Serine	P^m^	[49, 50]
OG-25	-	54734^WS^	-	-	-	-	-	Serine	P^m^	[34]
OG-26	-	43917	-	ATEG_06406^W^	-	NFIA_072360	Afu5g14610	Serine	P^m^	
OG-27	AN2555	56161^WS^	AO090010000534	ATEG_09537^WS^	AFL2G_11692	NFIA_035860	Afu2g03510^WS^	Serine	P^m^	[39, 51]
OG-28A	-	55665^WS^	AO090166000084^WS^	-	AFL2G_09418^WS^	NFIA_102320	Afu4g14000	Serine	P^m^	[52]
OG-28B	-	52700^WS^	-	-	-	-	-	Serine	P^m^	[52]
OG-29	AN5442	52603	AO090103000332	ATEG_03401	AFL2G_12064	*NFIA_059500* ^W^	Afu6g13540	Serine	P^m^	[34]
OG-30	AN7231^WS^	56689^WS^	AO090102000079^WS^	ATEG_10012^WS^	AFL2G_09533^WS^	NFIA_092750^W^	Afu2g17330^W^	Serine	P^m^	[39]
OG-31	AN1426^WS^	214460^WS^	AO090103000026^WS^	ATEG_00024^WS^	AFL2G_12331^WS^	NFIA_096830^WS^	Afu1g00420	Serine	P^m^	[53]
OG-32	AN10030	140344	AO090020000517	ATEG_06546^W^	AFL2G_10813	NFIA_078120	Afu5g09210^W^	Serine	P^m^	[40]
OG-33	AN2237^WS^	192619	AO090701000220^WS^	ATEG_09343^WS^	AFL2G_05864^WS^	NFIA_079940^W^	Afu5g07330	Serine	P^m^	
OG-34	AN7159^W^	211032^WS^	AO090011000235^WS^	ATEG_02150^WS^	AFL2G_05009^WS^	NFIA_029950^WS^	Afu4g03490^WS^	Serine	P^m^	[54]
OG-35	-	-	AO090701000579^WS^	-	AFL2G_06196^WS^	-	-	Serine	P^m^	
OG-36	-	-	-	-	-	NFIA_031000	Afu7g08350^WS^	Serine	P^m^	
OG-37	AN2818^W^	180130	AO090103000478	-	AFL2G_11938	-	-	Serine	C	
OG-38	*AN1182* ^WS^	208263	AO090038000317	ATEG_00287	*AFL2G_07674* ^W^	NFIA_014730	Afu1g10910	Serine	P^m^	[38, 55]
OG-39	AN2366^WS^	-	AO090023000609^WS^	ATEG_05749	AFL2G_04440^WS^	-	-	t-serine	A	[56]
OG-40	AN5558^WS^	203039	AO090003001036^WS^	ATEG_03900^W^	AFL2G_01995^WS^	NFIA_104430^W^	Afu4g11800	t-serine	A	[42, 57]
OG-41	AN0224	35620	*AO090023000428* ^W^	*ATEG_05010* ^W^	*AFL2G_04274* ^W^	NFIA_057190	Afu6g11500	t-serine	A	
OG-42	AN5129	181371	AO090012000995	*ATEG_10178* ^W^	*AFL2G_03855* ^WS^	NFIA_081390^W^	Afu1g07440	Ubiquitin	U	[57–59]
OG-43A	AN0687	207954	*AO090012000528* ^WS^	*ATEG_00551* ^WS^	*AFL2G_03425* ^WS^	NFIA_012010	*Afu1g13490* ^W^	Ubiquitin	U	[45]
OG-43B	*AN2000* ^WS^	214265^W^	*AO090003001182* ^WS^	ATEG_00694	AFL2G_01863^W^	NFIA_105720	Afu4g10350	Ubiquitin	U	[60]
OG-44	AN7254	205183	AO090102000107	*ATEG_10033* ^W^	*AFL2G_09558* ^W^	NFIA_092420	Afu2g17110	Ubiquitin	U	[61]
OG-45	AN4016	52026	AO090003000947	*ATEG_03809* ^W^	AFL2G_02080	NFIA_020680	Afu1g04040	Ubiquitin	U	[62]

The proteases found in both WB and SBP are marked^WS^, the ones only found in WB are marked^W^ and the ones only found in SBP are marked^S^. Putative non-extracellular proteins detected by proteomics are in italics. Orthologous proteases are clustered and mentioned in the first column. Absence of orthologs in each species are resembled by “-”.

*Abbreviations:* *B* Bestatin, *P* Pepstatin, *E* E-64/L-cysteine, *E* ^*d*^ EDTA, P^m^: AEBSF/DFP/PMSF, *C* Calyculin A, *A* Aprotinin/Antipain, *U* Ubiquitinyl hydrolase 1, *T-serine*, trypsin-like serine.

From all identified proteins, 93 were found on both WB and SBP, while 38 were found uniquely on WB and only two (dipeptidyl-peptidase Afu4g09320 [51, 63] and neutral protease I NFIA_099860) were found uniquely on SBP. Twenty-five out of these 133 identified proteases were not predicted to be extracellular according to our combined SCL predictions. Some of them may be secreted through alternative (non-classical) secretion systems, as suggested for the spermidine synthase (AO090012000528) from *A. oryzae*.

**Figure 2:**
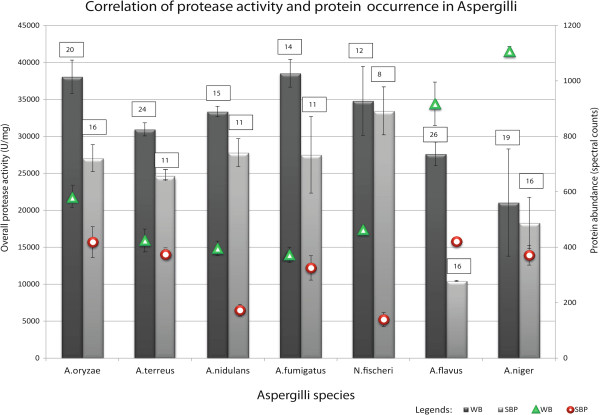
**Correlation of protease occurrence, abundance and activity in Aspergilli on WB or SBP.** While growing on 2 different crude substrates (on 30°C and sampled at 72 h), the protease occurrence, abundance and the enzyme activity of seven tested *Aspergillus* strains show a general positive correlation. In WB more occurrences of proteases with higher abundance have been identified than in SBP, so as the enzyme activities. The protease abundance is presented in this figure by spectral counts, the amount of identified proteins which is presented in the figure by framed numbers. Legends: WB and SBP: protease activity in wheat bran and sugar beet pulp, respectively; WB^P^ and SBP^P^: Protease abundance in wheat bran and sugar beet pulp, respectively.

While comparing proteomics-confirmed protein productivities to enzymology-identified protease activities, a strong correlation was found: WB generally induced more proteases than SBP with all tested *Aspergillus* species taking protein occurrence, abundance and enzyme activities all in consideration (Figure 2).

Intriguingly, contradictions were also found when delving deeper into the protease production profile of individual species. For example, *A. terreus* has the second largest amount of proteases identified in WB (24) whereas only 11 proteins were found in SBP. However, the protease activity in SBP was only around 20% less than in WB (Additional file 4). In *N. fischeri* a lower than average number of proteins was detected by proteomics, but the protease activity was amongst the highest of all species. In *A. fumigatus* only 3 additional proteins (15% extra quantity by spectral counts) were identified in WB compared to SBP, but the overall protease activity in WB was 40% higher. These findings demonstrate that the total protease activity does not only depend on the total production of proteases. This is likely due to the fact that the enzyme assays measure the combined activity of the available proteases. As they have different specific activities, the total activity is not equal to the sum of the protein amount. For instance, high abundance of a protease with a low specific activity may affect the overall protease activity less than moderate abundance of a protease with a high specific activity.

### Closer examination of the produced protease activity using protease inhibitors

In order to elucidate the extracellular protease composition in more detail, a series of inhibitor specificity tests was performed. Most of the proteases that were identified in Aspergilli could be classified into the following major groups: amino, aspartic, metallo and serine. Based on literature, the main inhibitor of each group was Bestatin [64, 65], Pepstatin [66–68], Ethylenediaminetetraacetic acid (EDTA) [69, 70] and phenylmethanesulfonylfluoride (PMSF) [71], respectively (Table 2, Additional file 4). These inhibitors were added to the supernatants and protease activities were compared to those without inhibitors (Additional file 5).In general, for all samples protease activity was found to be inhibited predominantly by PMSF (43.48-67.12% decrease of activity). Lesser inhibition of activity was detected with EDTA (1.56-40.05%), Bestatin (1.29-14.92%) and Pepstatin (2.24-28.40%) (Figure 1B).

For PMSF inhibition the ratio of decrease was similar in all species (55 ± 12%), even though *A. niger* has the lowest overall protease activity and *N. fischeri one of* the highest (Figure 1 and Additional file 5). No significant difference of PMSF inhibited activities was found between WB and SBP in *A. oryzae, A. nidulans, A. terreus*, *N. fischeri* and *A. niger*. Although the occurrence and abundance of serine proteases were different in the samples, PMSF inhibited around half of the protease activity in all samples (Figure 1B, Additional file 5). Nevertheless, some of the prevalently produced serine protease clusters may be responsible for at least half of total enzyme activity in these species regardless of carbon source differences. Examples could be OG-30 that contains the lysosomal Pro-Xaa carboxypeptidase ProtA (56689) [39, 47], OG-31 that contains the dipeptidyl peptidase II (214460) [39], OG-33 that contains the carboxypeptidase CpyI (AO090701000220) [53] and OG-34 that contains the tripeptidyl-peptidase TppA (AO090011000235) [54] (Table 2).

**Figure 3:**
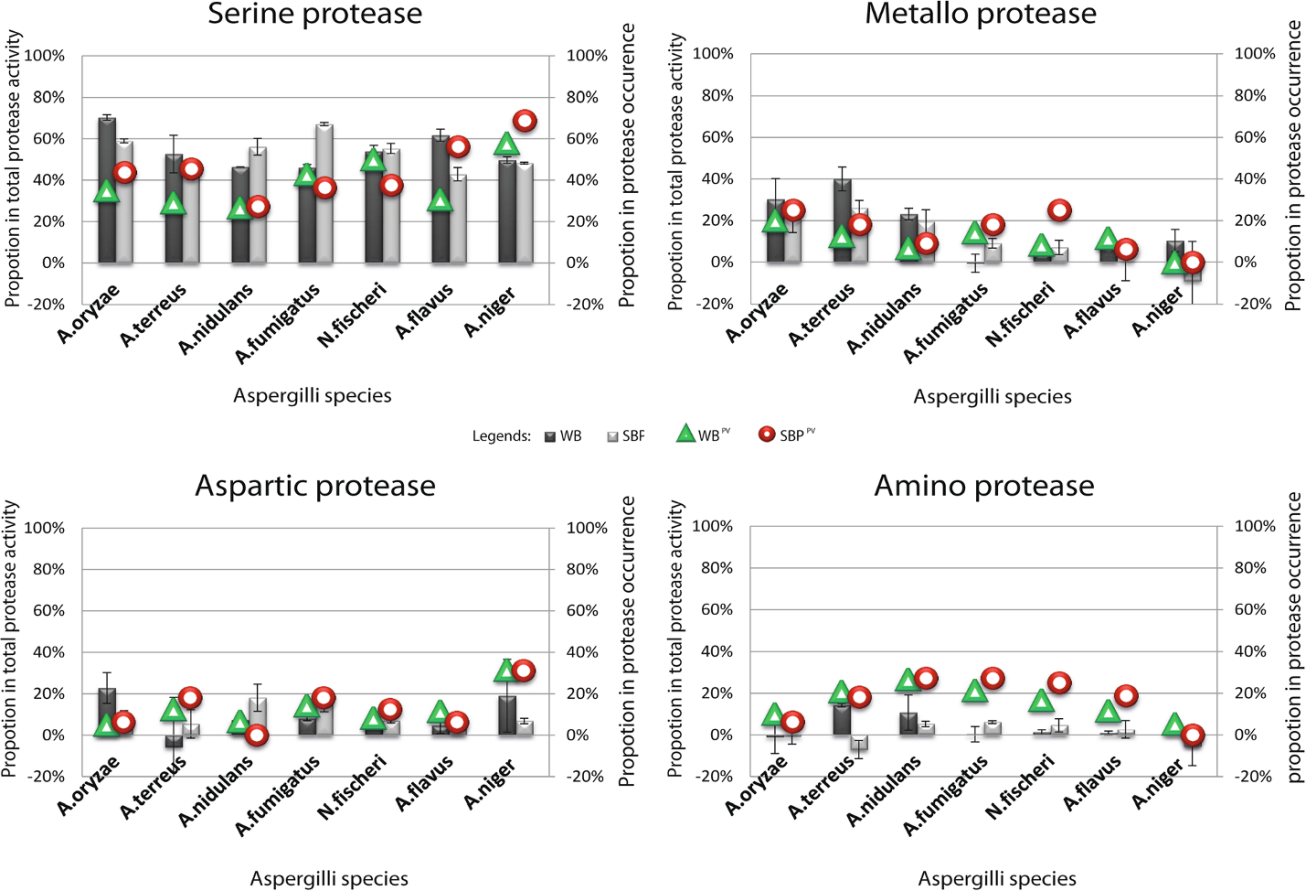
**Correlation for each subgroup of protease by occurrence and activity in Aspergilli on WB or SBP (growing on 30°C and sampled at 72 h).** The protein occurence is presented by the percentage of serine, metallo, aspartic and amino proteases in all proteomics-identified proteases, respectively. The proportion of serine, metallo, aspartic and amino proteases is presented by the percentage of inhibited enzyme activity by adding PMSF, EDTA, pepsatin and bestatin (PercentageDroppedActivity = [1- (activity after adding inhibitor/original activity without inhibitor)]% as in Figure 2). The occurrence of corresponding protease in the spectrum was calculated by AmountSpecificProtease/AmontTotalProtease%. Legends: WB and SBP: Proportion of specific protease activity in the spectrum while growing on wheat bran and sugar beet pulp, respectively; WB^PV^ and SBP^PV^: percentage of protease occurrence in wheat bran and sugar beet pulp, respectively.

In contrast, *A. fumigatus* and *A. flavus* showed noticeable inhibition differences depending on the growth substrate. In *A. flavus* inhibition of serine proteins on WB showed a 50% higher effect than that on SBP. The opposite was observed for *A. fumigatus* where SBP seemed to promote more serine-protease activity than WB (Figure 1B and Figure 3).

EDTA was the second best inhibitor, but a large variation of inhibited activity was detected (1.56-40.05%). *A. terreus* and *A. oryzae* showed the highest activity inhibition in WB samples. *A. nidulans* also showed a significant inhibition effect (~20%), though only a small difference between WB and SBP was detected. The enzyme activity in these species did not show strong correlation to their protease profiles. In *A. terreus* minor amounts of metallo proteases were identified by the proteomics experiments. The spectral counts in WB were comparatively lower than in SBP, even though the activity inhibition was much higher in WB than in SBP, suggesting higher specific activity of metallo proteases present in WB. Alkaline protease AN7962 [43] was the only metallo protease detected in *A. nidulans* cultures by proteomics. The spectral counts of this protein in WB were two-fold higher than in SBP. EDTA showed an equal effect on this protein with both substrates. In *A. oryzae* the main metallo proteases identified were neutral protease I (AO090011000036), neutral protease II (AO090010000493) and the leucine aminopeptidase (AO090011000052) [45]. They showed higher total abundance in WB than SBP, and the inhibition effects confirmed this.

Among all species, *A.oryzae* and *A.niger* showed the highest activity inhibition when pepstatin was added to the supernatant produced with WB, indicating the presence of aspartic proteins in these species.In *A. niger* Aspergillopepsin A (PepA, 201655) [42] was found to be the most dominant protease, with a four-fold higher abundance in WB than in SBP.

Bestatin mainly inhibits the activity of amino-protease/peptidases. Adding Bestatin to the supernatants showed minor inhibition of protease activities (<10%). This was intriguing because a rather high presence of amino proteases was identified in *A. terreus*, *A. nidulans*, *A. fumigatus, A. flavus* and *N. fischeri* by proteomics. The putative aminopeptidase OG-4 [33, 36] (AN8445, AO090003000354, ATEG_09137, AFL2G_02631, NFIA_001250, and Afu3g00650) was the most abundant amino protease regardless of the carbon source in all species except *A. niger* (Figure 3).

## Discussion

We have performed sets of heterogeneous tests on *Aspergillus* species using two complex substrates as carbon sources, aiming to construct a snapshot of fungal life that reflects the variation in protease productivity in different species. In contrast to commonly reported genome-scale protease analysis results [27, 48, 62], besides comparative genomics and proteomics we also included the analysis of enzymatic measurements, which provided further elucidation on the composition of extracellular protease spectra.

By comparative genomics, a rather even distribution (around 3%) of putative proteases was detected in *Aspergillus* genomes despite genome size variations (Table 1). Among species, the proportions of proteins in each specific subgroup were also consistent, namely 25 ± 1% serine, 18 ± 1% metallo, 8 ± 1% amino and 5% aspartic proteases. Further ortholog clustering revealed only a very low number of extracellular “orphan” genes (9 putatively extracellular genes that have no homologs in the other six species included in this analysis). In fact, more than 60% of the extracellular putative proteases clusters were found to be ubiquitous by containing at least one gene per species. Moreover, the major extracellular protease regulator PrtT was also found to have a single presence per *Aspergillus* genome (except for *A. nidulans*) [51, 72–75]. This might have brought assumptions that in during evolution, moderate divergence of protease genes has occurred in this genus since most of the encoding genes were well conserved at sequence level and only a small number of species-specific genes was identified. If this hypothesis applies, the production rate of extracellular proteases in all *Aspergillus* species should follow the distribution of encoded genes and have similar influence of the regulator prtT, meaning even protein count and quantitative measurement should be detected by proteomics. However, large variations in protein occurrence and abundance were found, indicating more profound mechanisms might be playing important roles.

For example, *A. flavus* and *N. fischeri* contain almost identical percentages of putative extracellular proteases in the genomes, but when cultivated on the same carbon sources a double amount of proteases and even higher abundance were identified in *A. flavus*. Should the protease productivity in Aspergilli follow the distribution of protease encoding genes, the production of each specific subgroup of protease would be consistent among categories and species. Indeed at least one semi-ubiquitous protease ortholog group of genes were identified for each sub-category of proteases on at least one of the substrates, such as OG-4 (lap2 amino protease, AN8445) [36], OG-9 (pepE aspartic protease, AN2903) [41], OG-16 (neutral metallo protease I, AO090011000036) [45] and OG-30 (ProtA serine protease, 56689) [39]. Moreover, a larger number of serine proteases were identified in all species, which correlates with the serine protease encoding genes being the largest subgroup of proteases in Aspergilli. However when quantitative measurements (abundance) were taken into account this correlation was absent because the most abundant individual proteases were never in the serine group, neither did the sum of abundances of the total serine group per species make this the dominant group (Additional file 4). In *A. flavus* (AFL2G_02631), *A. fumigatus* (Afu3g00650), and *A. oryzae* (AO090003000354) the most abundant protease belong to the amino protease group, while in *A. nidulans* (AN7962) and *A. terreus* (ATEG_04941) the most abundant proteases were metallo proteases. In the other species aspartic proteases (201655 and NFIA_073740) were more abundant. Taking *A. niger* as an example, the highest amount of serine proteases were indeed identified in the supernatant. However, based on comparative genomics the second most abundant group should be the metallo proteases, but no metallo protease was detected by proteomics on either substrate, which could possibly indicate that some of the proteases of the other classes also require metal ions [76, 77]. The second most abundant group detected in *A. niger* were the aspartic proteases, including *pepA* (213261) [34], *opsA* (211797) [40] and *opsB* (53364) [39]. This demonstrated that even on the same substrate protease occurrence and abundance in *Aspergillus* species can differ significantly.

Although in industrial applications the productivity of proteases usually refers to the production rate of proteases per time per unit, in this study we aim to construct a snapshot of Aspergilli life style which reflects the protease production mechanisms, therefore the productivity measurements of proteases did not only include the occurrence or abundance of proteins but also the enzyme activities.

Summarizing the comparison results of genomics, proteomics and enzymology tests, a general trend was detected. WB induced higher total protease activity, richer proteomics profiles and more protein abundance than SBP. This strongly suggests that in Aspergilli, carbon source difference is the most important factor that influences protease productivity (see Additional file 6 for monosaccharide composition of WB and SBP and [78] for the composition of amino acids). This was further confirmed by the fact that using glucose (minimal medium +3% glucose) or glucose plus casein (minimal medium +1% glucose + 1% casein) only low protease induction could be detected in *A. nidulans* while sampled at the same time point as the WB or SBP cultures (data not shown).

While outside the scope of this study, it should be mentioned that it has been frequently reported that proteases are largely produced upon environment-induced cell lysis/damage [38], especially with sugar or nitrogen depletion [33, 36, 79, 80]. In our analysis, WB-based substrates showed higher protease activity as well as profiles than SBP-based substrates. This may indicate that WB cultivation resulted in a faster growth rate and earlier sugar depletion, and has therefore promoted an earlier production of proteases [36, 81]. To further reveal the mechanisms behind *Aspergillus* protease productivity, aspects such as sugar consumption and fungal growth rate should be taken into account in future studies.

Besides amino, aspartic, metallo and serine proteases, a certain amount of ubiquitin and trypsin proteases were also detected by proteomics. The specificity of these proteases was not tested due to the unavailability of inhibitor kits. Although very low abundance was found for these proteins, these proteins may also take part in the total extracellular protease activity in Aspergilli.

Other factors may also cause variability between individual *Aspergillus* species. pH has been reported to be one of these factors [46, 82, 83] and some of the data of this study supports this assumption. For example, AN6888 (*pepA*) has been reported to be an acidic protease [42] and was not detected in *A. nidulans* (pH 7 on WB and 8 on SBP). In contrast, the ortholog of this protein in *A. niger* (201655) had high abundance (pH = 5-6) [39, 49, 75, 80].

Finally, even though 6 well known protein SCL predictors were employed in order to guarantee the accuracy of extracellular protease prediction, improvements could still be made for secretome prediction. Among all six used tools the prediction rate varied largely. The WoLF-PSORT prediction fitted best with the proteomics results, while Multi-LOC was most different from this (data not shown). Interestingly, although with low area abundance 25 proteases were detected extracellularly by proteomics that lack a translocation signal peptide. Most of them were found in *A. flavus* and *A.terreus* (7 proteins each species), 3 were found in *A. oryzae* and the rest disseminated among the other species. If this was not caused by cell lysis or leakage, these proteins can be considered as indications of alternative secretion systems in Aspergilli. Hardly any of these proteins were correctly predicted by the SCL predictors we used. Hence, this study may also be of value as a testing or training set to improve currently existing prediction methods.

## Conclusions

We have performed a series of *in silico* and biological experiments to gain understanding of protease production in Aspergilli. According to the results of comparative genomics *Aspergillus* species contain a similar proportion of protease encoding genes with serine proteases as the biggest group. The proteomics and enzymatic experiments generally confirm this composition, as serine proteases indeed make up the largest subcategory in the protease spectrum across the species. Furthermore, taking carbon source differences into account, wheat bran resulted in a higher induction of proteases than sugar beet pulp. An interesting variation of total protease activity, composition of the protease spectrum, and their abundance were observed between the species. The broadest set of proteases was found in *A. flavus*, while the highest overall protease abundance was found in *A. niger*, and the highest protease activity was detected for *A. fumigatus* in wheat bran and for *N.fischeri* in sugar beet pulp. It is very likely that even cultivated in an identical environment, the tested *Aspergillus* species were experiencing different physiology when sampled at the same time point. Concerning the high protein sequence conservation level (1E-20, sequence coverage 85%) among clustered proteases, it is likely that the variation of protease productivity is caused by more complicated mechanisms such as gene regulation related to environmental changes by carbon source differences [35, 44, 46] but not by enzymatic differences between the orthologous proteases themselves.

## Methods

### Genome mining, clustering and extracellular protein prediction

The genome sequences were extracted from AspGD [28] (version May 2014). Used genome information is listed in Table 1.

The pre-calculated protease clusters in AspDG were retrieved from the Aspergillus10-way-comparative database. Additional homologs were added to the clusters by homolog searches using majority vote of BLASTP [84], Jaccard [29] (cutoff E-value e-20 and alignment coverage 85%) and OrthoMCL[85] (E-value 1E-10, inflation level 1 and sequence coverage 40%) results. Gene models were double checked with manual curation combining literature searches.

Six protein subcellular localization (SCL) predictors, Phobius [86], SignalP [87], PrediSi [88], CELLO [89], MultiLOC [90] and WoLF-PSORT [91, 92] were used to predict the extracellular proteases. Default settings of each SCL predictor were used, with the species parameter as “Eukaryotic” or “Fungi”. Majority votes were applied to combine the results of each SCL prediction.

### Protease inhibitor information extraction and other bioinformatics analysis

The specific enzyme inhibitor information was retrieved by AspGD gene annotation repository and literature researches. Protein functional domain prediction was performed by HMMER v.3.0 [93] using the complete Pfam-A and Pfam-B models [94] (data retrieved from Pfam database, version November 2012) with the trust cutoff and the gathering cutoff. The resulting Pfam predictions were pooled.

### Strains and media

The fungal strains used in this study are listed in Table 1. All strains were grown on Malt Extract Agar and incubated at 30°C for 3–4 days until good sporulation had occurred. Spores were harvested by gentle agitation in 10 ml ACES (acid buffer) and solutions were taken into sterile tubes. Twenty times dilution of each solution were counted using a haemocytometer (Burker-Turk) under microscope (Axioplan, Zeiss). Liquid media was prepared in 250 ml conical flasks containing 50 ml Minimal medium (MM) [95]. Five different culture conditions were prepared for the determination of protease activity in different mediums. Below substrates were added into 250 ml conical flasks containing 50 ml MM and a) 1% wheat bran, b) 1% wheat bran +1% glucose, c) 1% sugar beet pulp, d) 1% glucose + 1% sugar beet pulp and e) 1% glucose + 1% casein. All prepared media were autoclaved at 121°C for 20 min. For each strain, sterile liquid culture media were inoculated with 5× 108 spore/ml in 250 ml erlenmeyer flask and incubated for 72 h at 30 0C on a shaker at 250 rpm for the production of proteases. During the growth of fungi, 2 ml of aliquots were taken from cultures at 48 h, 72 h and 96 h. Those were centrifuged and used for all the experiments. Cultures were established in duplication for biological repetition and triplicated for technical repetition. The pH of most samples on was 7 except for *A. nidulans* on SBP (pH = 8) and *A. niger* on WB (pH = 5-6) and SBP (pH = 4-5).

### Protease activity assay

A pilot experiment was performed on *A.nidulans* growing on WB, WB + Glc, SBP and SBP + Glc and protease activities were measured on 48, 72 and 96 h post-inoculation. From the analysis the best day with highest protease activity was found to be day 3 (72 h post-inoculation, Additional file 7).

For all experiments, protease activities of the cultures were measured after 72 h post-inoculation in liquid-state fermentation. 2 ml samples were taken from flasks and centrifuged at 14000 × ρ for 10 min (Eppendorf Centrifuge, 5417R). Supernatant was separated after centrifugation and stored at -20°C until the measurements of protease activity.

The protease activity assay was performed according to the procedures mentioned in protocol of Pierce Fluorescent Protease Assay Kit (Kit number: 23266, Pierce Biotechnology, Thermo Scientific, USA). The levels of protease activity in the supernatants of 7 strains over 72 h were compared using a fluorescein isothiocyanate (FITC)-labeled casein assay according to the manufacturer’s instructions. Fluorescence of the samples were measured by optical density (OD) using the plate reader (Fluostar Optima, BMG LABTECH) with excitation at 485 nm and emission at 530 nm to determine protease activity. The enzyme activity was expressed as micromoles of trypsin released per minute per milligram of total protein in culture filtrate (unit: U/mg, 1 μmol trypsin min-1).

pH 7.2 was required for the Pierce in light of the TBS solution stability. A pilot experiment was performed testing this kit on pH 4, 6, and 8 (Additional file 8). According to the result of this test, pH 6 was selected for protease activity measurements.

### Inhibition of proteases

Protease inhibitors were prepared to give final concentrations of 50 mM for PMSF and EDTA (Sigma), 1 mM for Pepstatin A and Bestatin (Sigma) as instructed by the manufacturer.

2 μL protease inhibitors were added into the assay mixture and incubated for 60 min at room temperature prior to performing the assay. Culture supernatants treated alone was used as negative control. Each assay was performed in triplicate. All measurements were performed under pH 6.

### Neutral carbohydrate composition

Neutral carbohydrate composition of wheat straw and sugar beet pulp was analysed according to Englyst [96] using inositol as an internal standard. Samples were treated with 72% (w/w) H2SO4 (1 h, 30°C) followed by hydrolysis with 1 M H2SO4 for 3 h at 100°C and the constituent sugars released were derivatised and analysed as their alditol acetates using gas chromatography (GC).

### Proteomics experiments

#### Protein digestion

Protein from 3 ml of incubation medium was precipitated with cold TCA/Acetone. Protein sample determination was carried out with the RCDC kit assay (BioRad, Mississauga, Ont). Five ug. of protein was incubated in 100 mM ammonium bicarbonate, 0.1% AALS II (Morgantown, WV) and 5 mM dithiothreitol for 30 min. followed by the addition of Iodoacetamide to a final 25 mM concentration and incubated for an additional 30 min at 37 Deg. C. 200 ng of trypsin was added to each sample and the solution totaling 70 ul was incubated for 18 hr at 37 deg C. The digestion solutions were acidified with trifluoroacetic acid (1% final) then put through two rounds of desalting using C18 ziptips™ (Millipore, Billerica, MA). Eluted peptides were dried in a SpeedVac and resuspended in a 60 ul solution of 5% ACN, 0.1% FA and 4fmol/ul of predigested Bovine Serum Albumin (Michrom, Auburn, CA) used as an internal standard.

#### LC-MS/MS analysis

Five ul of peptide digest was loaded onto 15 cm × 75 μm i.d PicoFrit column (New Objective, Woburn, MA) packed with Jupiter 5 μm, 300 Å, C18 resin (Phenomemex, Torrance, CA) connected in-line with a Velos LTQ-Orbitrap mass spectrometer (Thermo-Fisher, San Jose, CA). Peptide separation was done using a linear gradient generated by an Easy-LC II Nano-HPLC system (Thermo-Fisher) using a mixture of solvent A (3% ACN:0.1% FA) and solvent B (99.9% ACN:0.1%FA). The gradient started at 1% B, was set to reach 27% B in 85 min, ramped to 52% B in 15 min and 90% B in 5 min then held at 90% for 5 min.

The mass spectrometer used was a Velos LTQ-Orbitrap (Thermo-Fisher, San Jose, CA). The capillary voltage on the nanospray source was adjusted to get the best spraying plume at 10% B and typically ranged from 1.9 to 2.1 kV. MS survey scan spanning the 350 to 2000 m/z range was done at 60000 resolution. The top 10 doubly, triply or quadruply charged ions with intensity higher that 5000 counts were considered candidates to undergo CID MS/MS fragmentation in the LTQ-Velos ion trap. Quantification was based on MS precursor ion signal using the precursor ion detection workflow from Proteome Discoverer Quant 1.3 (Thermo-Fisher). Briefly, extracted ion chromatograms were generated to compute the peptide area value associated to each identified precursor ion. A Protein Area value is subsequently calculated as the average of the three most intense, distinct, peptides assigned to a protein. Protein area values were expressed as a fold value of the protein area value calculated for Bovine Serum Albumin (BSA) which was spiked as an internal standard in each individual sample. For spectral count-based comparisons, the number of assigned spectra for each protein was reported as a fold value of the total number of spectra assigned to BSA in each sample.

#### Bioinformatics data processing

LC-MSMS data was processed using Proteome Discoverer Quant 1.3 (Thermo-Fisher) and spectral data was searched against Aspergillus protein databases downloaded from the Aspergillus Genome Database (AspGD). Search parameters used were 0.80 Da for fragment ion tolerance of and 10.0 ppm for parent ion tolerance, fixed iodoacetamide cysteine modification and variable methionine oxidation. Quantification was based on MS precursor ion signal using the precursor ion detection workflow from Proteome Discoverer Quant 1.3 (Thermo-Fisher). Briefly, extracted ion chromatograms were generated to compute the peptide area value associated to each identified precursor ion. A Protein Area value is subsequently calculated as the average of the three most intense, distinct, peptides assigned to a protein. Protein area values were expressed as a fold value of the protein area value calculated for Bovine Serum Albumin (BSA) which was spiked as an internal standard in each individual sample. For spectral count-based comparisons, the number of assigned spectra for each protein was reported as a fold value of the total number of spectra assigned to BSA in each sample. Confidence filters were applied to satisfy a 1% FDR at the Peptide and Protein level. Protein grouping was applied so as to satisfy the principles of parsimony. The normalized protein areas of a protein were used as the measurement of abundance level of the protein. The abundance of a protein represents the productivity of the protein in an organism under that specific circumstance while measured. The areas are also used as the measurement of protease productivity.

The unique counts of peptides to each identified protein were used as evidences of the occurrence of the protein. For any protein that has more than one uniquely mapped peptide it is considered occurred in the supernatant. The total amount/number of proteases in a sample was calculated by the sum of proteins which have more than one uniquely mapped peptide.

The mass spectrometry proteomics data have been deposited to the ProteomeXchange Consortium (http://proteomecentral.proteomexchange.org) via the PRIDE partner repository [97] with the dataset identifier PXD000982.

### Availability of supporting data

The mass spectrometry proteomics data have been deposited to the ProteomeXchange Consortium (http://proteomecentral.proteomexchange.org) via the PRIDE partner repository [97] with the dataset identifier PXD000982.

## Electronic supplementary material

Additional file 1: **The putative protease clusters in seven Aspergilli species.** The subcellular localization prediction, protease family classification, Pfam domain prediction and functional annotation of each protein are listed. The proteins found in the original AspGD are marked bold. (XLS 607 KB)

Additional file 2: **The "Orphan" proteases in 7 Aspergilli.** The "Orphan" proteases in 7 Aspergilli, together with their subcellular localization prediction, protease family classification, Pfam domain prediction and functional annotation. (XLS 38 KB)

Additional file 3: **The subcellular localization prediction on putative proteases.** Using majority vote the compartment of the proteins was predicted by 6 different tools. (XLS 416 KB)

Additional file 4: **The proteomics identification of proteases.** Description of data: the proteomics identification of protease in Aspergilli with SBP and WB respectively. Both ion count and spectral counts are provided. (XLS 86 KB)

Additional file 5: **The original protease activity measurements without and with inhibitors.** Description of data: Two batches of biological replicates were measured with triplicated technical replicates of each sample, the triplicates were corrected by blanco and the mean/standard deviation values are listed in the table. (XLS 50 KB)

Additional file 6: **The monosaccharide composition of wheat bran and sugar beet pulp.** (XLS 10 KB)

Additional file 7: **The time series enzyme activity measurements of** ***A. nidulans*** **on day 2, 3 and 4.** The protease activity and inhibition tests were measured under pH 7.2 (recommended by manufacturer), on 48, 72 and 96 h post-inoculation. (XLS 28 KB)

Additional file 8: **The pH optimization pilot test with protease activity measurements.** The protease activity was measured under pH 7.2 (recommended by manufacturer), 4, 6 and 8. pH 6 was used to perform further experiments for less bias was caused. (XLS 12 KB)
